# A New Complexity Layer: DNA Methylation and the Predictive Impact of Epigenetic Tests

**DOI:** 10.3390/ijms27031611

**Published:** 2026-02-06

**Authors:** Giorgio Ladisa, Francesca Montenegro, Angela Picerno, Alessio Nigro, Antonella Cicirelli, Alessandra Stasi, Marco Fiorentino, Paola Pontrelli, Loreto Gesualdo, Fabio Sallustio

**Affiliations:** 1 Department of Interdisciplinary Medicine, University of Bari Aldo Moro, 70124 Bari, Italy; g.ladisa13@phd.uniba.it (G.L.); antonella.cicirelli@uniba.it (A.C.); 2 Department of Precision and Regenerative Medicine and Ionian Area (DiMePRe-J), University of Bari Aldo Moro, 70124 Bari, Italy; francesca.montenegro@uniba.it (F.M.); angela.picerno@uniba.it (A.P.); anigro571@gmail.com (A.N.); alessandra.stasi@uniba.it (A.S.); marco.fiorentino@uniba.it (M.F.); paola.pontrelli@uniba.it (P.P.); loreto.gesualdo@uniba.it (L.G.); 3 PersonGene, s.r.l., 70124 Bari, Italy

**Keywords:** DNA methylation, epigenetics, inflammation, cancer, metabolic disease, epigenetic tests

## Abstract

The increasing complexity of disease mechanisms challenges accurate diagnosis, prevention, and early risk stratification. Beyond genetic predisposition, epigenetic regulation—particularly DNA methylation—represents a dynamic molecular interface linking environmental exposures, metabolic imbalance, inflammation, and disease development. DNA methylation is the most extensively studied epigenetic mechanism and plays a central role in controlling gene expression across physiological and pathological conditions. In this review, we provide an integrated overview of DNA methylation biology and its involvement in inflammatory, metabolic, and oncological diseases, with a specific focus on pathways related to chronic inflammation and oxidative stress. We summarize evidence demonstrating how aberrant methylation patterns contribute to disease initiation and progression, highlighting recurrent epigenetic signatures affecting key regulatory genes. In parallel, we discuss current and emerging technologies for DNA methylation analysis, ranging from targeted methylation-specific assays to next-generation sequencing-based approaches, including nanopore adaptive sampling. Finally, we explore the translational potential of DNA methylation-based tests as predictive and preventive tools, emphasizing their ability to identify disease-associated molecular alterations before clinical onset. Overall, this evidence supports the integration of epigenetic profiling into future precision medicine strategies aimed at early risk assessment, prognosis refinement, and personalized prevention.

## 1. Introduction

Studying and characterizing diseases has become increasingly complex. The same pathological condition can now be investigated from multiple, complementary perspectives, requiring a multidisciplinary approach. Specifically, disease analysis now spans anatomical and morphological features, functional alterations assessed through biomarkers in different biological matrices, and molecular and genomic layers in which disease-associated genotypes may act as diagnostic markers. This stepwise, hierarchical view reflects the principles of systems pathology, which integrates multi-omics data and spatial heterogeneity to capture the complexity of disease mechanisms [1]. However, an additional layer of complexity was introduced by epigenetics in the early 2000s, providing new insights and previously unknown mechanisms that further enrich the pathophysiological multifaceted nature of a disease [2,3]. Epigenetics is the study of inheritable modifications in gene function that do not involve changes to the DNA sequence. An epigenetic trait is a stably heritable phenotype that results from chromatin-based changes that regulate gene expression. These traits are reversible, modifiable, and can be inherited both somatically and through the germline [4,5]. However, they are influenced by random environmental changes that can be physical or chemical stressors, impacting the regulatory and enzymatic network underlying these epigenetic processes. Furthermore, mutations in the genes responsible for encoding the epigenetic machinery can cause shifts in the epigenetic landscape. Consequently, the gene expression profile may undergo temporary or long-term alterations. For instance, loss of function in Ten-Eleven Translocation (TETs) protein family, which play a key role in DNA demethylation, have been shown to disrupt epigenetic regulation and gene expression programs [6,7,8].

These modifications are controlled by molecular processes and factors, such as DNA methylation (DNAm), histone modifications, ATP-dependent chromatin remodelling complexes, and non-coding RNAs, which regulate gene activation or silencing [9,10,11,12].

## 2. DNA Methylation and the Interplay Between Histone Modification, Chromatin Remodelling and Non-Coding RNAs

The most studied and well-known epigenetic mechanism is DNAm, reflecting gene expression regulation. DNAm can remodel chromatin and make it inaccessible for the transcription process. This chemical modification consists of the addition of a methyl group (CH3) to the carbon 5 of a cytosine, usually located in 5′-cytosine-phosphate-guanine-3′ (CpG) dinucleotides. The distribution of CpG dinucleotides is asymmetrical throughout the genome, and their accumulation preferentially occurs upstream or within promoter regions, defining the so-called CpG Islands (CGIs) [13]. Indeed, CGI hypermethylation leads to transcriptional silencing, regulating gene expression through a differential methylation pattern both in early life stages and adulthood. During embryogenesis, the methylation pattern is largely erased during the first cell divisions and is later re-established by de novo DNA methyltransferases (DNMT’s), which direct tissue-specific gene expression programs [14]. In adulthood, the CpG dinucleotide methylation pattern is restored during each replication by maintenance DNMT’s, both in differentiated cells and during the self-renewal of adult stem cell populations. In both cases, fine epigenetic regulation ensures the preservation of cellular and tissue-specific identity and functionality [15]. However, methylation has the feature of being reversible due to the action of demethylating enzymes [16]. This reversibility is crucial for the role of epigenetic modifications, and it can be modulated by different signals, leading to a changed gene expression [17] and cellular differentiation. In the human genome, there are approximately 30,000 unmethylated CGIs that ensure a potentially active chromatin configuration, which is typical of housekeeping genes. The DNAm pattern plays a key role in cellular differentiation, and the epigenetic profile modulates gene expression across various cell types, during developmental stages, and in both health and disease states [17]. Cytosine methylation can significantly alter temporal and spatial gene expression as well as chromatin remodelling. It is increasingly clear that these epigenetic mechanisms play a vital role in regulating metabolic phenotypes and other complex diseases, thereby representing a promising therapeutic target [18,19,20,21,22,23].

DNAm, while the most extensively studied epigenetic mechanism to date, is not the only epigenetic process influencing gene expression [18].

Post-translational modifications made on nucleosomes (formed by H2A-H2B histone tetramer and two H3-H4 histone dimers, such as acetylation, phosphorylation, methylation, SUMOylation, and ubiquitination, can alter the structure or the ionic charge in these proteins, modifying their interaction with DNA [24]. Consequently, such modifications can regulate gene expression by modulating the accessibility of transcription factors to specific DNA regions, highlighting the dynamic interplay between histone proteins and nucleic acids in transcriptional regulation [25,26].

Notably, although DNA methylation and histone acetylation represent distinct epigenetic mechanisms, an important regulatory crosstalk exists between these two systems, which can reinforce both repressive and permissive states of gene expression [27].

Histone acetylation, mediated by histone acetyltransferases (HATs), occurs on lysine residues of histone tails and neutralizes their positive charge, thereby weakening histone–DNA interactions. This process leads to a more open and accessible chromatin configuration, ultimately promoting gene transcription [28]. In contrast, histone deacetylation, mediated by histone deacetylases (HDACs), restores the positive charge of histones, resulting in chromatin compaction and transcriptional repression [29,30],

Several studies have demonstrated that these epigenetic events can cooperate and reciprocally influence each other in establishing transcriptionally active or repressive chromatin states [31]. The presence of 5-methylcytosine within gene promoters can directly impair the binding of transcriptional activators; however, a substantial component of its repressive effect is mediated through the recruitment of chromatin corepressors that remove acetyl groups from neighboring histones [32].

For instance, methyl-CpG-binding protein 2 (MeCP2) recognizes methylated cytosines via its methyl-CpG-binding domain (MBD) and, through its transcriptional repression domain (TRD), recruits a corepressor complex containing SIN3 transcription regulator family member A (Sin3A) and HDAC1/2 [33]. This interaction results in local histone deacetylation and chromatin remodeling toward a compact conformation, thereby inhibiting transcription [31]. Similarly, other members of the MBD protein family, such as MBD2, are integral components of repressive complexes including NuRD (nucleosome remodeling and deacetylase), which bind methylated CpG regions and promote histone deacetylation, leading to transcriptional silencing of the associated genes [32].

Repressive interactions also occur between DNA methylation and histone methylation. DNMT1 and DNMT3A/3B can associate with the histone methyltransferase suppressor of variegation 3–9 homolog 1 (SUV39H1), which catalyzes the methylation of histone H3 at lysine 9 [34,35]. Through this mechanism, DNA methylation and H3K9 methylation are deposited in a coordinated manner, reinforcing a stable, long-term inert chromatin state [36]. Accordingly, heavily methylated genomic regions typically exhibit a loss of histone H3 and H4 acetylation and a concomitant gain of repressive histone marks such as H3K9me3 or H3K27me3 [36].

However, this crosstalk also operates in the opposite direction and can promote gene expression. The acetylation status of histones actively influences DNA methylation patterns. In general, chromatin enriched in acetylated histones is associated with hypomethylated DNA: transcriptionally active regulatory regions, such as promoters of expressed genes, tend to be simultaneously acetylated at the histone level and unmethylated at the DNA level [37]. Experimental evidence supports a causal relationship between these processes. Pharmacological inhibition of HDACs, which increases global histone acetylation levels, has been shown to induce DNA demethylation [38]. For example, in cell lines treated with butyric acid or trichostatin A (TSA)—a potent HDAC inhibitor—a reduction in 5-methylcytosine levels at promoter-associated CpG islands and the transcriptional reactivation of previously silenced genes have been observed [39].

An additional key factor underlying this bidirectional regulation is the selectivity of DNA methyltransferases as influenced by histone marks. Certain activating histone modifications can physically prevent the recruitment of DNA methyltransferases to chromatin [40]. A well-characterized example is the trimethylation of histone H3 at lysine 4 (H3K4me3), a hallmark of active promoters. DNMT3A and DNMT3B contain regulatory domains that preferentially recognize H3 tails unmethylated at lysine 4; when H3K4 is already methylated, as in transcriptionally active genes, DNMT activity at the corresponding promoter is inhibited [41]. Consequently, the presence of activating histone marks, including H3K4me3 as well as acetylation marks such as H3K9ac and H3K27ac, contributes to the protection of CpG islands from aberrant DNA methylation, thereby sustaining gene expression [42,43,44].

The bidirectional crosstalk between DNA methylation and histone signaling allows these epigenetic layers to reinforce each other in either repressing or promoting gene expression [45].

Beyond these chemical modifications of histones, other processes contribute to chromatin modification. Specific ATPase complexes (reg. SWI/SNF, ISWI, CHD, and INO80) participate in remodelling chromatin, enabling the transition between condensed and relaxed states [46]. Additionally, histone phosphorylation contributes to this regulation. Phosphorylation can influence the methylation status on histones such as H3K9 and H3K27, which are involved in several diseases [47,48,49]. This interplay between phosphorylation and methylation represents an important crosstalk that further complicates the regulation of gene expression.

**Figure 1:**
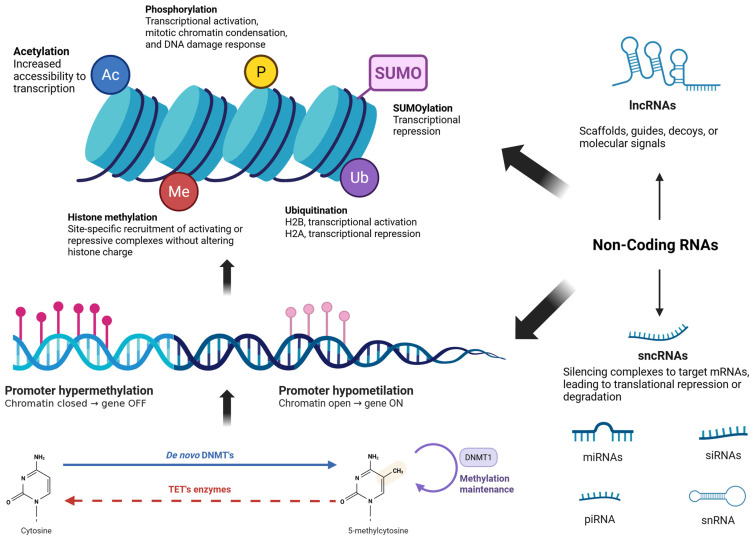
Epigenetic regulation of gene expression. Gene expression is regulated by interconnected epigenetic mechanisms, including DNA methylation, histone post-translational modifications, chromatin remodeling, and non-coding RNAs. DNA methylation at CpG islands, particularly in promoter regions, is associated with transcriptional repression when hypermethylated and with permissive chromatin states when unmethylated. Histone modifications modulate chromatin accessibility through site-specific recruitment of activating or repressive complexes. Long and small non-coding RNAs further fine-tune gene expression by guiding and modulating epigenetic and transcriptional machinery by affecting histone modifications and DNA methylation (black arrows).

There is also a bidirectional interaction between DNA methylation and non-coding RNAs (ncRNAs) representing an additional layer of epigenetic regulation. These ncRNAs are classified into Small non-coding RNAs and Long non-coding RNAs (lncRNAs) [50,51]. They play diverse roles in regulating gene expression, including forming protein scaffolds, as observed with lncRNAs [52], or directly binding to mRNA to suppress its translation [53] (Figure 1).

On one hand, DNA methylation at regulatory regions (e.g., promoters) can alter ncRNA expression—typically silencing them when promoter regions become hypermethylated. On the other hand, several ncRNAs (including microRNAs, lncRNAs, and piwi RNAs) modulate the DNA methylation machinery by influencing the expression or recruitment of DNMTs and other epigenetic regulators [54].

This kind of crosstalk establishes epigenetic gene–feedback loops that are frequently disrupted in diseases such as cancer, chronic inflammatory disorders [55].

In cancer, the interplay between ncRNAs and DNA methylation is evident in both directions: ncRNAs are subject to epigenetic regulation, and they themselves modulate DNA methylation patterns [54]. A fitting example is the epigenetic silencing of tumor-suppressor miRNAs via promoter hypermethylation. This has been documented in various malignancies. For example, in breast cancer, cancer-initiating cells display hypermethylation of the miR-34c promoter, resulting in downregulation of this miRNA, which normally targets NOTCH4 and suppresses epithelial-to-mesenchymal transition (EMT) [56]. Conversely, some tumor-suppressor miRNAs actively reshape the epigenome by targeting DNA methyltransferase transcripts. A well-studied example is the miR-29 family in lung cancer, which significantly downregulates DNMT3A and DNMT3B, thereby promoting demethylation and reactivation of multiple tumor suppressor genes [57]. This epigenetic reprogramming yields measurable antitumor effects, including reduced tumor growth and restoration of suppressive functions, and forms the basis for experimental strategies in miRNA-based epigenetic therapy [57,58]. Alongside miRNAs, lncRNAs also participate in these regulatory dynamics. Due to their ability to interact with DNA, RNA, or proteins, lncRNAs often act as “guides” or “scaffolds” for epigenetic complexes [59]. Several lncRNAs have been shown to physically associate with DNMT1, DNMT3A/B, or chromatin-modifying enzymes such as Enhancer of Zeste Homolog 2 (EZH2), G9a, and TET proteins, thereby directing them to specific gene loci [60]. For example, in renal progenitor cells, the lncRNA HOTAIR can regulate the CD133 stem cell marker expression, self-renewal properties and the secretion of the anti-aging protein Klotho, inducing the trimethylation of the histone H3K27 and the epigenetic silencing of the cell cycle inhibitor p15 [52].

In an inflammatory context, such as post-infarction cardiac fibrosis, another mechanism has been described involving *NEAT1* [61]. Under hypoxic stress, activation of DNMT3A leads to hypermethylation of the NEAT1 promoter, downregulating its expression. This reduction in NEAT1 levels triggers activation of the NLRP3 inflammasome and pyroptosis in cardiac fibroblasts, contributing to inflammatory cell death and fibrotic tissue deposition [62]. Inhibition of DNMT3A—either pharmacologically or genetically—prevents NEAT1 methylation, preserves its expression, and attenuates both pyroptosis and subsequent myocardial fibrosis [62].

## 3. DNA Methylation in Inflammatory, Cancer, and Metabolic Diseases

Inflammation is a protective response of the body to tissue damage caused by physical, chemical, or biological agents aimed to eliminate the source of injury. This mechanism, shaped by natural selection, can cause prolonged tissue damage or functional impairment, particularly when the inflammation becomes chronic [63]. Inflammatory cells are recruited to the site of injury, activating pro-inflammatory pathways through signalling molecules such as pathogen-associated molecular patterns (PAMPs), damage-associated molecular patterns (DAMPs), and cytokines, leading to an exacerbation of the inflammatory response [63]. If not resolved promptly, this can progress to chronic inflammation, often resulting in functional decline of the tissue. Inflamed tissue is characterized by a rich milieu of signalling and pro-inflammatory molecules, along with a slight acidification of the extracellular environment [64,65].

All these alterations and stimuli act as epigenetic stressors, potentially modifying the methylation patterns of genes both locally and systemically, thereby affecting their expression [66]. Furthermore, these inflammation-induced epigenetic changes can have long-term effects in both tissue functionality and overall organismal homeostasis, particularly influencing the activity of DNMTs and TETs enzymes, which are responsible for adding and removing methyl groups on DNA [67]. Altered DNMT activity can lead to hypermethylation or hypomethylation in specific genes [68]. Hypermethylation in promoter regions can silence tumor suppressor genes or other genes involved in resolving inflammation, while hypomethylation can activate oncogenes or pro-inflammatory genes, contributing to pathological processes such as tumorigenesis or chronic inflammation [69]. In fact, the epigenetic relationship between inflammation and a tumorigenic microenvironment is notably close, and aberrant methylation patterns of key inflammation-related genes contribute to the establishment of a pro-tumorigenic microenvironment [70].

**Figure 2:**
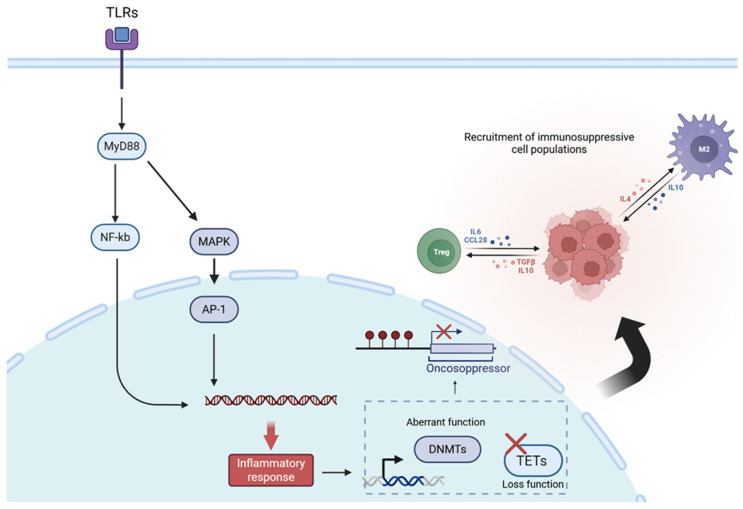
Mechanism of long-term chronic inflammation impact in DNA methylation, and overall tumorigenic microenvironment. The continuous and chronic activation of inflammatory pathways, together with persistent oxidative stress, leads to the loss of function and dysregulation of key epigenetic regulatory enzymes such as DNMTs and TETs. Consequently, epigenetic homeostasis is disrupted, affecting the normal expression of proto-oncogenes and tumor suppressors, contributing to the recruitment of immunosuppressive cell populations (black arrow) and the development of an immune-evasive microenvironment.

Mechanistically, this process unfolds through a cascade of molecular events. Initially, acute inflammation—triggered by conditions such as obesity, diabetes, or autoimmune disorders—progresses into a chronic state. This persistent inflammation leads to the sustained activation of key signaling pathways, including NF-κB, JAK/STAT, MAPK, TLR, and MyD88. The associated oxidative stress, primarily mediated by reactive oxygen species (ROS), promotes epigenetic alterations, inducing aberrant DNMTs activity and concurrent loss of TETs enzyme [71]. These changes result in a disrupted equilibrium between pro- and anti-inflammatory signaling, characterized by the overexpression of proto-oncogenes and silencing of tumor suppressor genes [72]. Ultimately, this molecular imbalance fosters the development of a pro-tumorigenic microenvironment, facilitated by the recruitment of immunosuppressive cell populations, such as regulatory T cells (Tregs) [73] (Figure 2).

Such epigenetic alterations can promote persistent inflammation, disrupt epithelial homeostasis, and ultimately facilitate malignant transformation. A frequently observed epigenetic feature in several cancers is the presence of loci known as Methylated in Tumor (MINT), which are genomic regions characterized by tumor-specific hypermethylation [74]. These MINT loci are often located within or near CpG islands associated with tumor suppressor genes. MINTs are considered excellent epigenetic biomarkers, as they have the potential to enable early cancer detection, prognostic assessment, and may also assist in optimizing therapeutic strategies [75]. A recent study showed that in hepatocellular carcinoma (HCC) a broad panel of genes undergoes significant promoter hypermethylation during the early stages of tumor development. Hypermethylation of *ZNF334*, a transcription factor involved in regulating cell differentiation, cell proliferation, apoptosis, tumor transformation, and anticancer defense and *CDKN2A*, a tumor suppressor locus located on 9p21 and one of the most complex and important in oncology, have been implicated in the disruption of cell cycle control [76]. Moreover, several MINT loci, including MINT1, MINT2, MINT12, and MINT31, have been identified as frequently methylated not only in HCC but also across multiple cancer types, suggesting a broader oncogenic role [74]. In the context of chronic intestinal inflammation, the hypermethylation of key regulatory genes can drive tissue toward an oncogenic trajectory. In samples from patients with Crohn’s disease, several strongly hypermethylated promoters have been identified, including *sFRP1*, *sFRP2*, and *sFRP5*, all known antagonists of the Wnt signaling pathway, as well as *TFPI2*, *Sox17*, and *GATA4* [77].

Nowadays, numerous studies performing Whole-Genome Bisulfite Sequencing (WGBS) have reported several instances of hypomethylated genes. In HCC, *CDC20* has emerged as a key hypomethylated gene, with both its up-regulation and promoter hypomethylation progressively increasing with tumor stage [78]. Moreover, in lung adenocarcinoma, a multi-omic analysis showed that aberrant DNA demethylation leads to the activation not only of cancer-germline genes, but also of entire clusters of genes normally restricted to other tissues [79].

In prostate cancer, the loss of *LKB1* promotes global DNA hypomethylation, and it has been shown that TET inhibitors could be useful in counteracting this demethylation [80]. Finally, a study using WGBS on murine and human pancreatic organoids at various tumor stages revealed multiple differentially methylated regions [81].

In the context of metabolic diseases, numerous epigenome-wide association studies (EWAS) have emerged in recent years, identifying specific DNAm alterations in key metabolic and inflammatory pathway genes. A recent systematic review of EWAS conducted on blood, adipose, liver or pancreatic islets identified 130 differentially methylated genes compared to healthy controls. Among the most frequently reported genes across the 32 analyzed studies were *TXNIP*, *ABCG1*, *PPARGC1A* (*PGC-1α*), and *PTPRN2*. All these genes were previously associated with genetic risk for type 2 diabetes (T2D) [82]. Furthermore, the same meta-analysis highlights how methylation levels of genes such as *IGFBP2*, *MSI2*, *FTO*, *TXNIP*, *SREBF1*, *PHOSPHO1*, *SOCS3*, and *ABCG1* are significantly associated with the onset of T2D or hyperglycemia [82].

Metabolic syndrome (MetS) is a multifactorial condition characterized by central obesity, insulin resistance, hypertension, and dyslipidemia. Recent studies suggest that MetS is also associated with a distinct epigenetic profile. Several epigenetic markers identified in type 2 diabetes (T2D) or obesity have also been reported in MetS. In an epigenome-wide meta-analysis conducted on peripheral blood from over 2300 individuals (Korean population), 40 CpG sites and 27 differentially methylated regions (DMRs) were significantly associated with MetS. Notable among these were *TXNIP*, a regulator of glucose and lipid metabolism; *ABCG1*, involved in lipid transport; and *TANK*, an adaptor in the NF-κB signaling pathway [83].

Additional epigenomic studies have identified CpG markers associated with adiposity and body mass index (BMI). One of the most consistent findings is the involvement of the gene HIF3A. Multiple EWAS have shown that increased BMI correlates with hypermethylation at specific intragenic sites of Hypoxia-Inducible Factor 3, Alpha subunit (*HIF3A*) in blood. This is an oxygen-sensitive transcription factor belonging to the HIF system, which regulates the cellular response to hypoxia. Beyond *HIF3A*, several other genes have emerged, including *CPT1A*, which encodes a key enzyme in fatty acid oxidation [84].

Many of these obesity-associated epigenetic alterations affect genes involved in inflammatory pathways, insulin/leptin signaling, and lipid metabolism, indicating that obesity is accompanied by widespread epigenetic remodeling across multiple systems [85]. Epigenetic evidence suggests that MetS is not merely the sum of individual metabolic risk factors, but rather has a distinct molecular signature at the level of DNAm. In the future, specific blood-based epigenetic biomarkers could help identify individuals with or at risk for MetS at an early, potentially reversible stage.

## 4. Inflammation and Epigenetic Changes

**Figure 3:**
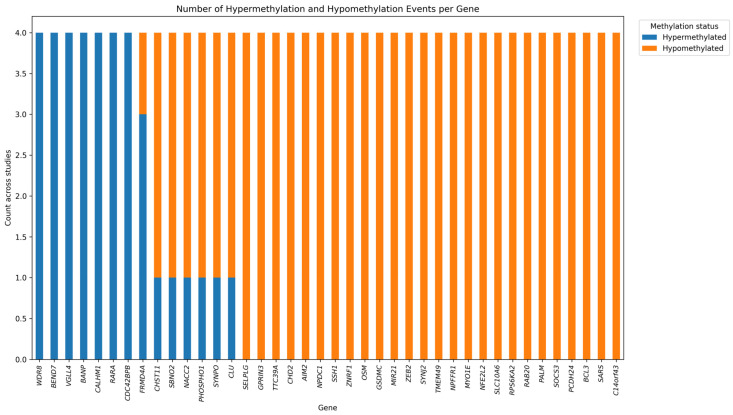
Bar plot showing the number of hypermethylation (orange) and hypomethylation (blue) events per gene across four independent studies of CRP-associated low-grade inflammation. The *y*-axis indicates the number of studies reporting a given methylation direction (maximum = 4). Genes most frequently identified in four CRP-related low-grade inflammation studies are shown on the *x*-axis, while the *y*-axis reports their frequency and methylation status. Hypermethylated genes are shown in red and hypomethylated genes in blue.

One of the methodologies currently employed in studies to understand how chronic inflammation modulates DNAm has primarily focused on associating inflammatory markers in plasma with the methylation levels of DNA in white blood cells. Among the various inflammatory biomarkers, the most used is CRP (C-reactive protein), which is particularly valuable for assessing ‘low-grade’ chronic inflammation. Interestingly, the DNA methylation of the same forty genes was found to correlate with CRP in four different studies (Table 1). Some genes were found to be involved with coherent hyper- or hypomethylation status across the studies, while others had discordant methylation levels. Moreover, most of these genes were consistently hypomethylated in all examined papers (Figure 3). These four studies demonstrate that, in large and multi-ethnic cohorts, CRP-associated CpG sites tend to exhibit consistent effects across ethnic groups. Collectively, they highlight that CRP-associated DNA methylation changes measured in the same biological matrix are consistently associated across different ancestries, considering the wide diversity in observed populations in the four studies [86,87,88,89].

Wielscher M. et al. [90] conducted a comprehensive meta-analysis combining data from 25 cohorts to investigate the association between the DNAm signature of chronic low-grade inflammation and CRP levels. The extensive sample size enabled the evaluation of DNAm patterns across diverse ethnic groups. The analysis revealed that a substantial portion of CRP-associated DNAm was driven by BMI, whereas a smaller fraction of the CpG signature was influenced by smoking. Many of the CpG islands were located near the Polymerase II subunit A (*POLR2A*) gene, highlighting how this gene is influenced by methylation. Furthermore, consistent with other studies, the methylation of the *AIM2* gene—a key component of the cytosolic inflammasome AIM2 that is activated in the presence of non-self-DNA or aberrant DNA- appears to be sensitive to CRP levels, showing a significant decrease in the presence of elevated markers of chronic inflammation and accompanied by an increased expression of NOD2, a cytosolic NLR [90]. In addition, acute stressors, such as lipopolysaccharides (LPS) or reactive oxygen species (ROS), can induce significant changes in the methylation profile. Specifically, these acute stressors can reduce the expression of DNMT1, leading to the upregulation of genes like *IL-6* and *IL-8*, along with heightened activity in pathways such as NF-κB and MAPK. This is because DNMT1 plays a key role in controlling the methylation of promoters associated with these pathways and inflammatory biomarkers [91].

Chronic low-grade inflammation is not the only factor able to modify the DNA methylation of specific genes. Heritable, tissue-specific phenotypic changes can also be driven by environmental stressors that significantly impact gene expression. Such stressors include air pollutants (e.g., PM2.5, ozone), heavy metals (lead, cadmium), pesticides, cigarette smoke, ultraviolet and ionizing radiation, unbalanced diets, alcohol consumption, obesity, chronic infections, psychological stress, early-life trauma, gut dysbiosis, and pharmaceuticals or endocrine-disrupting chemicals (such as Bisphenol A and phthalates). Prenatal factors, including maternal malnutrition or stress, can also shape the offspring’s epigenome through early programming (Table 2).

## 5. Epigenetic Changes in Oxidative Stress-Related Genes

Many of these stressors and their associated pathological effects significantly contribute to the increase in oxidative stress. Different genes are involved in oxidative stress regulation, such as superoxide dismutase 2 (*SOD2*), Glutathione peroxidase 1 (*GPX1*), and nuclear factor erythroid 2-related factor 2 (*NRF2*), as well as genes of major inflammatory pathways like *IL-6*, *NF-κB*, and *TNFα*.

### 5.1. SOD2

Superoxide Dismutase 2 (*SOD2*) encodes a mitochondrial enzyme that plays a crucial role in cellular defense against oxidative stress. It catalyzes the dismutation of superoxide anions (O_2_^−^•), which are highly reactive products of mitochondrial respiration, into hydrogen peroxide (H_2_O_2_) and molecular oxygen (O_2_). By limiting the accumulation of superoxide radicals, SOD2 preserves mitochondrial function, prevents oxidative damage to proteins, lipids, and DNA, and contributes to the regulation of redox-sensitive signaling pathways involved in apoptosis, inflammation, and cellular senescence. A recent study revealed that in bleomycin-induced pulmonary fibrosis models, the downregulation of *SOD2*, mediated by genetically induced DNMT3A loss, promotes mitochondrial ROS production and lung fibroblast proliferation, contributing to the development of pulmonary fibrosis [99]. Clinical and experimental studies have also reported methylation changes in the promoter region of *SOD2*. For instance, prenatal exposure to PM10 and NO2 has been associated with *SOD2* promoter hypermethylation in umbilical cord DNA, with levels proportional to maternal exposure [100]. Conversely, in a clinical trial involving women who had undergone breast cancer surgery, an aerobic exercise intervention led to an approximately 20% reduction in *SOD2* promoter methylation [65,101].

### 5.2. GPX1

Glutathione Peroxidase 1 gene (*GPX1*) encodes a selenium-dependent enzyme that plays a critical role in cellular antioxidant defence. It catalyzes the reduction of hydrogen peroxide (H_2_O_2_) and organic hydroperoxides to water and their corresponding alcohols using reduced glutathione (GSH) as an electron donor. By detoxifying peroxides, *GPX1* helps maintain redox homeostasis, protects cells from oxidative damage, and modulates signaling pathways related to apoptosis, inflammation, and mitochondrial function. In mice subjected to exercise was shown the alteration of key antioxidant enzymes (SOD2, TRX1, PRDX3, AND GPX1) in skeletal muscle. Notably, only GPX1 exhibited exercise- and dyslipidemia-associated changes in DNAm. Dyslipidemia induced hypermethylation of a CpG site in the second exon of *GPX1*, while exercise transiently reduced methylation at this locus [102]. In experimental models of oxidative stress, the promoter region of *GPX1* has been shown to be epigenetically sensitive to environmental conditions. For instance, in cultured cardiomyocytes exposed to Advanced Glycation Endproducts (AGEs), an increase in *GPX1* promoter methylation is observed, leading to decreased gene expression. Conversely, selenium or treatment with the DNA methyltransferase inhibitor 5-azacytidine significantly reduces this promoter methylation [103].

### 5.3. NRF2

The Nuclear Factor Erythroid 2-Related Factor 2 (NRF2 or NFE2L2) is a master transcriptional regulator of the cellular antioxidant response. Under basal conditions, NRF2 is sequestered in the cytoplasm by its inhibitor KEAP1 and targeted for proteasomal degradation. Upon oxidative stress or exposure to electrophilic agents, NRF2 is stabilized and translocates to the nucleus, where it binds to antioxidant response elements (AREs) in the promoter regions of its target genes, initiating their transcription [104]. This gene contains a prominent CpG island within its promoter region, and physical exercise, such as running, has been shown to induce demethylation of the *NRF2* promoter, which correlates with restored *NRF2* expression [105]. An interesting in vitro study aimed at investigating the carcinogenic properties of nano-silica (Nano-SiO_2_), revealed a specific mechanism of *NRF2* epigenetic regulation by mapping two CpG-rich regions within the *NRF2* promoter. Following repeated treatment in 16HBE and BEAS-2B cell line, with Nano-SiO_2_, methylated alleles in one of these regions disappeared while the other region remained methylated. This epigenetic shift was accompanied by a marked increase in *NRF2* mRNA and protein levels, as well as in its downstream targets, *HO-1* and *SOD1* [106]. In the transgenic adenocarcinoma of mouse prostate (TRAMP) mouse model, the *NRF2* promoter—including part of the first exon and intron—was found to be highly hypermethylated (~96%) in tumor tissue compared to normal prostate tissue (~4%) [107]. In mice fed with a high-fat diet (a model of hepatic steatosis) and in HepG2 cells exposed to high glucose, the *NRF2* promoter becomes hypermethylated, leading to lipid accumulation and downregulation of antioxidant genes. Treatment with 5-azacytidine or resveratrol reverses these effects by demethylating the *NRF2* promoter, restoring *NRF2* expression and that of its target genes (e.g., *NQO1*, *SOD*), and ultimately reducing oxidative stress and steatosis [108].

### 5.4. IL-6

IL-6 (Interleukin-6) is a pleiotropic pro-inflammatory cytokine involved in both innate and adaptive immune responses. It is produced by multiple cell types, including macrophages, T and B lymphocytes, fibroblasts, and endothelial cells, in response to infection, stress, or tissue injury. IL-6 regulates the acute-phase inflammatory response, stimulates hepatic production of acute-phase proteins CRP, and influences lymphocyte differentiation. Dysregulation of *IL-6* expression has been associated with chronic inflammatory, autoimmune, and neoplastic diseases [109].

Several studies have reported alterations in *IL-6* promoter methylation in response to oxidative stress, with direct consequences on *IL-6* expression. In human cell cultures, excessive reactive oxygen species (ROS) or antioxidant deficiency induces epigenetic changes at the *IL-6* gene. For instance, zinc deficiency—a condition known to generate oxidative stress—has been shown to reduce *IL-6* promoter methylation in human lymphoblastoid cell line (THP-1 cells), which was associated with a marked increase in *IL-6* expression compared with control cells [110]. Similarly, direct exposure of cells to hydrogen peroxide rapidly increases *IL-6* expression through activation of NF-κB at the *IL-6* promoter region [111], suggesting that oxidative stress may relieve epigenetic constraints and promote *IL-6* transcription.

In the same study, murine models further support these observations. In aged mice subjected to a zinc-deficient diet, hypomethylation of the *IL-6* promoter was observed. Notably, these effects were pronounced in aged animals but were much less evident in young mice, indicating that aging—together with its associated burden of chronic oxidative stress—renders the Il6 promoter more susceptible to demethylation and transcriptional activation in response to oxidative insults [110].

Clinical and observational evidence further supports associations between exposure to oxidative factors and *IL-6* methylation. A relevant example is cigarette smoking, which is known to induce systemic oxidative stress. Cumulus cells (oophorus cumulus cells) collected from smoking women exhibited significantly lower methylation levels at the *IL-6* promoter compared with those from non-smokers [112].

In patients with ischemic heart disease, lower levels of *IL-6* methylation have been correlated with higher plasma IL-6 concentrations and an increased risk of coronary artery disease [113]. These findings support the concept that chronic oxidative stress—such as that associated with vascular inflammation and cigarette smoking—contributes to maintaining *IL-6* in an epigenetically active state (i.e., a less methylated promoter), thereby sustaining excessive IL-6 production in the circulation.

### 5.5. TNFα

TNFα (tumor necrosis factor alpha) or TNF is a prominent pro-inflammatory cytokine that is often released early under conditions of cellular and oxidative stress. It is a classical transcriptional target of NF-κB and a key mediator of systemic inflammation [114]. *TNFα* expression is subject to epigenetic regulation through DNA methylation, and oxidative stress can disrupt this control [115]. For example, an excess of pro-oxidant or pro-inflammatory nutrients in the diet may be reflected in the epigenome of the *TNF* gene. A nutrigenomic study reported that higher intake of ω-6 polyunsaturated fatty acids (n-6 PUFAs) was inversely associated with *TNF* promoter methylation in peripheral leukocytes [116] and pathological conditions characterized by intense oxidative stress and inflammation display specific alterations in *TNF* methylation [117]. An illustrative example is chemotherapy-induced oral mucositis. Methotrexate-based chemotherapy in pediatric patients causes tissue injury accompanied by massive release of ROS and pro-inflammatory cytokines, including TNFα. In a study involving pediatric oncology patients, DNA methylation of antioxidants and inflammatory genes, including *TNF*, was analyzed in oral mucosal cells. The results showed that in children who developed mucositis and subsequently recovered, the *TNF* promoter was frequently hypomethylated. In this context, however, hypomethylation of the *TNF* promoter and the resulting increase in TNFα production appeared to serve a recovery-related signaling function, highlighting the context-dependent nature of epigenetic modulation [118].

However, under conditions of chronic oxidative stress, *TNF* tends to undergo promoter hypomethylation, effectively creating an “ON-locked switch” that sustains excessive TNFα production and contributes to a deleterious state of chronic inflammation, as observed in obesity, atherosclerosis, and other oxidative stress-related diseases [117]. Conversely, promoter hypermethylation—induced, for example, by antioxidant-rich diets or supplementation—acts as a molecular brake, reducing TNFα expression and attenuating downstream TNFα-mediated inflammatory pathways [117,119].

### 5.6. NF-κB

**Figure 4:**
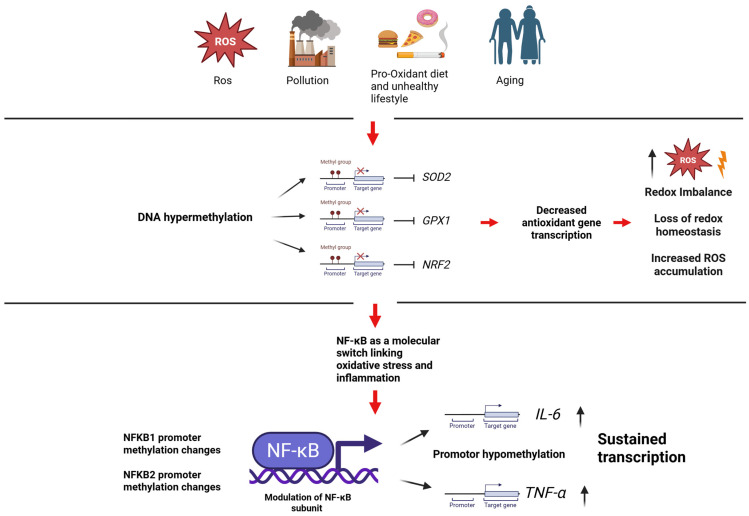
Epigenetic upstream–downstream link between oxidative stress and inflammation.

*NF-κB* is not a single gene but a family of transcription factors *p65* (*RelA*), *RelB*, *c-Rel*, *p105/p50* (*NF-κB1*), and *p100/52* (*NF-κB2*), and their associations coordinate the activation of inflammatory genes such as *IL6* and *TNF* [120]. Oxidative stress primarily influences NF-κB at the post-translational level by activating the IKK/IκBα signaling cascade, thereby promoting NF-κB nuclear translocation [111]. Emerging evidence indicates that epigenetic regulation of NF-κB-related genes can also change in response to prolonged oxidative stimuli, thereby modulating the amount of *NF-κB* available for transcriptional activation. A representative example comes from physical exercise, as regular physical activity modulates endogenous oxidative stress and exerts well-established anti-inflammatory effects [121]. In a pilot study involving elderly women, a 5-month Interval Walking Training (IWT) program induced significant increases in promoter methylation of *NFKB2* (*p52*) and, to a lesser extent, *NFKB1* (*p50*) in peripheral leukocytes [122]. Pharmacological or nutraceutical interventions that attenuate oxidative stress can also modify the epigenetic landscape of *NF-κB*-related genes. In a murine model of accelerated aging (SAMP8 mice, characterized by elevated oxidative stress), administration of the polyphenolic antioxidant resveratrol resulted in increased methylation of the *NRF2* and *NFKB1* genes in tissues from treated mice compared with controls [123]. In addition, cigarette smoking and environmental pollution—both major sources of environmental oxidative stress—are known to induce global changes in genomic DNA methylation (Figure 4). Large-scale epigenomic analyses have identified smoking-sensitive loci, including sites near *NFKB1*, which display altered methylation patterns in smokers [124,125]. However, in contrast to IL-6 and TNFα, NF-κB functions as a central regulator rather than a downstream effector; therefore, the impact of epigenetic modifications on *NF-κB* should be interpreted in terms of overall pathway activity. Hypermethylation of the *NFKB1* and *NFKB2* genes observed following antioxidant interventions and physical exercise tends to reduce the expression of these NF-κB subunits [126].

Together, these findings highlight the pivotal role of epigenetic regulation in modulating the antioxidant response under oxidative stress conditions. The genes *SOD2*, *GPX1*, and *NRF2* (*NFE2L2*) encode essential components of the mitochondrial and cellular redox defense system and are sensitive to both endogenous and environmental stimuli. These epigenetic modifications are context-dependent: while oxidative stress often induces hypermethylation and gene silencing, compromising antioxidant capacity, protective stimuli such as physical exercise or treatment with epigenetic modulators (e.g., resveratrol, selenium, 5-azacytidine) can reverse these marks, restoring gene expression [103,127,128]. Notably, *NRF2* emerges as a central redox-sensitive transcriptional hub whose methylation status is dynamically regulated in multiple pathological contexts, including cancer, steatosis, and environmental exposures. *SOD2* and *GPX1* are not only downstream targets of NRF2, but also directly subjected to methylation-dependent transcriptional control, further emphasizing the multilayered regulation of the antioxidant system.

Conversely, the cytokines IL-6 and tumor TNF-α play a central role in inflammatory processes, acting as key mediators of the inflammatory fire. Their production is tightly regulated by the transcription factor NF-κB, which serves as a primary regulator of pro-inflammatory gene expression [129]. A pivotal element linking cellular stress and inflammation is chronic oxidative stress. Excessive levels of reactive oxygen species ROS can sustain NF-κB in a state of persistent activation, thereby fueling a vicious inflammatory cycle [130]. ROS activate NF-κB, which in turn induces the continuous production of pro-inflammatory mediators (e.g., IL-6, TNF-α, COX-2), further exacerbating inflammation [131] (Figure 4). In parallel, cytokines such as TNF-α can further enhance ROS generation, creating a self-perpetuating feedback loop [132]. This mechanism contributes to the phenomenon of “inflammageing,” defined as a state of chronic, low-grade systemic inflammation associated with aging and numerous degenerative diseases [133]. However, accumulating evidence indicates that this pathological context can be modulated and attenuated through epigenetic regulation. Indeed, physical exercise, nutraceutical interventions, and a healthy lifestyle have been shown to promote a general hypermethylation of pro-inflammatory genes, thereby modulating the overall inflammatory tone [134].

The downstream outcome of this epigenetically reinforced signaling cascade is the establishment of chronic, low-grade inflammation and the development of inflammaging. Potential disruption of this pathological loop may be achieved through regular physical exercise, a sustainable and balanced diet, and targeted nutraceutical supplementation or pharmacological interventions, all of which have been shown to effectively modulate epigenetic marks and attenuate the oxidative–inflammatory circuit [135] (Figure 4).

Chronic oxidative stress and environmental exposures (e.g., ROS, air pollution, smoking, unhealthy lifestyle, and aging) induce epigenetic alterations, particularly DNA methylation. Hypermethylation of antioxidant genes (*NRF2*, *SOD2*, *GPX1*) impairs redox homeostasis, leading to ROS accumulation and activation of the NF-κB pathway. NF-κB acts as a central molecular switch, promoting the expression of pro-inflammatory cytokines (IL-6, TNF-α), whose sustained transcription is reinforced by promoter hypomethylation under chronic oxidative conditions. This self-perpetuating oxidative–inflammatory loop contributes to chronic low-grade inflammation and inflammaging, while lifestyle and therapeutic interventions may disrupt the circuit.

## 6. Diagnostic and Predictive Epigenetic Tests: Potential, Present, and Future

Given the profound impact of epigenetics on gene expression processes, it becomes evident that diagnostic tests capable of assessing a the methylation status of patient’s DNA are of fundamental importance. These tests are not only valuable for diagnosing disease itself—once the epigenetic contribution to the pathology is understood—but also for predicting disease onset. Indeed, aberrant methylation patterns often precede the clinical manifestation. This highlights the critical need to implement epigenetic tests into routine clinical practice, particularly for preventive purposes.

Current technologies for studying DNA methylation can be broadly divided into assays that require bisulfite conversion and those that do not. Another way to categorize these assays is based on whether they employ next-generation sequencing (NGS) or rely exclusively on the amplification of single genes or small multiplex panels.

Bisulfite-based assays include conventional PCR approaches such as methylation-specific PCR (MSP), methylation-sensitive high-resolution melting analysis (MS-HRM), and digital variants such as droplet digital PCR (ddPCR) [136]. In MSP, specific primers are employed, and the same gene is amplified multiple times: one primer set specifically amplifies the methylated sequence, while another targets the unmethylated sequence, both after bisulfite conversion [137]. This method is inexpensive, highly efficient, and straightforward to perform. However, there are limitations: in fact, conversion to bisulfite involves extremely harsh reaction conditions (acidic pH, temperature) that can degrade DNA. This makes analysis difficult when the DNA sample is already scarce and fragmented. To overcome these problems, alternative methods based on enzymatic conversion have been developed, such as Enzymatic Methyl-seq (EM-seq). Due to this methodology, the TET2 enzyme oxidizes 5-methylcytosine and 5-hydroxymethylcytosine (5mC/5hmC) into derivatives (e.g., 5-carboxylcytosine) that are protected from subsequent deamination, while the apolipoprotein B mRNA editing enzyme, catalytic polypeptide-like 3A (APOBEC3A) enzyme, selectively deaminates only unmodified cytosines, converting them into uracil. After PCR, the originally unmethylated cytosines therefore appear as thymines in the sequences obtained, while the methylated or hydroxymethylated cytosines remain read as cytosines, exactly as in the bisulfite protocol. Thanks to the absence of aggressive chemical conditions, the enzymatic strategy of EM-seq preserves DNA integrity and provides higher-quality methylation data than bisulfite [138]. Other limitations of bisulfite sequencing are the restricted output—typically one or a few genes—and the need for prior detailed knowledge of the target locus. By contrast, NGS-based bisulfite methods, which are also widely used but not in clinics, leverage second- and third-generation sequencing platforms. Techniques such as WGBS and reduced-representation bisulfite sequencing (RRBS) commonly employ sequencing platforms, providing read lengths of approximately 50–300 bp and representing the current standard for high-resolution methylation analysis [139]. For third-generation sequencing, PacBio and Oxford Nanopore offer protocols capable of producing long-read data, but the latter in particular offers the possibility of native nanopore methylation detection with adaptive sampling, which essentially represents a methodology that bypasses the need for bisulfite conversion, allowing direct generation of DNA methylation information. Adaptive sampling is a sequencing strategy that enables targeted enrichment of specific DNA regions instead of sequencing the entire genome [140]. The software is provided with a known reference sequence, and any DNA fragments that do not match or diverge substantially from this reference are actively rejected by reversing the pore voltage, ejecting the molecule from the nanopore.

This approach allows selective sequencing of defined regions of interest (ROIs), thereby markedly reducing costs and increasing overall sequencing efficiency [141]. Because the same principle can be applied to DNA methylation analysis, adaptive sampling is particularly advantageous for epigenetic studies: by supplying the software with a reference set of methylated CpG sites, the system can preferentially enrich these CpG islands, minimizing both the time and expense required compared with whole-genome methylation sequencing. In Utomo et al., a cohort of children affected by stunting—a condition resulting from chronic malnutrition—was studied using buccal swabs as a non-invasive DNA source. The primary aim was to evaluate adaptive sampling as a tool to detect epigenetic differences associated with chronic malnutrition and to investigate whether such nutritional stress leaves detectable marks on the epigenome. A panel of CpG regions was targeted, and children with stunting displayed a lower global 5mC level in buccal epithelial cells compared with well-nourished controls, indicating widespread hypomethylation in malnourished subjects. Furthermore, the authors identified a differentially methylated region (DMR) on chromosome 21at the *MIR6724* and *RNA45SN1* gene loci, revealing a previously unrecognized epigenetic association with stunting [142]. In Patel et al., the authors developed a rapid-classification workflow for central nervous system (CNS) tumors that leverages adaptive sampling. In this clinical setting, real-time intraoperative assessment of promoter methylation and molecular alterations is critical for accurate tumor typing. Using adaptive sampling, the workflow enabled simultaneous detection of somatic mutations and tumor DNA methylation status in a single assay, demonstrating how this strategy can transform oncologic diagnostics by delivering integrated genetic and epigenetic results directly from surgical tumor samples—without prolonged turnaround times or the need for highly specialized laboratory infrastructure [143]. Another example of clinical application is provided by the study of Yamada et al. In this work, the authors aimed to evaluate whether nanopore sequencing technology could be employed to detect abnormal DNA methylation patterns associated with imprinting disorders, specifically Prader–Willi syndrome (PWS) and Angelman syndrome (AS). Nanopore sequencing successfully identified the characteristic aberrant methylation signatures of both PWS and AS and was able to discriminate between normal and pathological phenotypes based on epigenetic alterations [144].

At present, commercially available assays and routine clinical practice rely primarily on classical methylation-specific PCR-based tests, which target a single gene or a small gene panel—provided that the specific epigenetic signature of interest is already well characterized [145,146]. Currently, no commercially marketed assays employ next-generation sequencing (NGS)-based methylation technologies; when NGS is used for DNA methylation detection, it remains largely confined to research settings. Nevertheless, such approaches are expected to enter clinical practice in the future, particularly given their high predictive potential. It is likely that predictive epigenetic tests will be developed and commercialized ahead of truly diagnostic epigenetic assays, providing risk estimates for metabolic alterations and states of oxidative stress or inflammation based on DNA methylation profiles. The literature already provides clear examples of specific DNA-methylation signatures with prognostic or “foretelling” power up to 15 years before disease onset [147,148].

However, robust, clinically validated methylation panels with proven predictive accuracy are not yet established and remain in the research or proposal stage. Consequently, widespread routine clinical implementation will require additional time, not least because most of these assays depend on NGS platforms, which currently remain costly.

Among emerging strategies, adaptive sampling with nanopore sequencing appears to offer a promising compromise between cost, efficiency, and turnaround time, but further protocol development and validation will be needed before it can be adopted in routine clinical diagnostics (Table 3).

## 7. Conclusions

In conclusion, accumulating evidence indicates that DNA methylation represents a key molecular layer bridging environmental stressors, chronic inflammation, oxidative imbalance, and disease susceptibility. Unlike static genetic variants, epigenetic marks are dynamic, context-dependent, and potentially reversible, making them particularly suitable for early risk stratification and preventive intervention. Even subtle, locus-specific methylation changes can significantly influence disease onset, progression, and chronic maintenance.

Accordingly, future diagnostic frameworks should move beyond genomics alone and incorporate epigenetic information to capture both the current molecular state and future disease trajectories. Advances in DNA methylation technologies—especially next-generation sequencing-based approaches—offer unprecedented opportunities to develop predictive tests with improved sensitivity and specificity. The integration of validated DNA methylation signatures into clinical practice may therefore represent a critical step toward truly predictive, preventive, and personalized medicine.

## Figures and Tables

**Table 1:** Methylation status per gene on four CRP-low grade inflammation different studies.

Gene	Lundin et al. [86]	Ligthart et al. [87]	Hillary et al. [88]	Chilunga et al. [89]
*C14orf43*	↓	↓	↓	↓
*SLC10A6*	↓	↓	↓	↓
*ZNRF1*	↓	↓	↓	↓
*SBNO2*	↓	↓	↑	↓
*AIM2*	↓	↓	↓	↓
*RPS6KA2*	↓	↓	↓	↓
*CDC42BPB*	↑	↑	↑	↑
*SARS*	↓	↓	↓	↓
*CHD2*	↓	↓	↓	↓
*NPFFR1*	↓	↓	↓	↓
*BEND7*	↑	↑	↑	↑
*BCL3*	↓	↓	↓	↓
*PCDH24*	↓	↓	↓	↓
*PHOSPHO1*	↓	↓	↑	↓
*SOCS3*	↓	↓	↓	↓
*PALM*	↓	↓	↓	↓
*RAB20*	↓	↓	↓	↓
*CHST11*	↓	↓	↑	↓
*RARA*	↑	↑	↑	↑
*CLU*	↑	↓	↓	↓
*WDR8*	↑	↑	↑	↑
*NFE2L2*	↓	↓	↓	↓
*NACC2*	↑	↓	↓	↓
*MYO1E*	↓	↓	↓	↓
*SYNJ2*	↓	↓	↓	↓
*TMEM49*	↓	↓	↓	↓
*CALHM1*	↑	↑	↑	↑
*ZEB2*	↓	↓	↓	↓
*SELPLG*	↓	↓	↓	↓
*GPRIN3*	↓	↓	↓	↓
*TTC39A*	↓	↓	↓	↓
*SYNPO*	↓	↓	↑	↓
*BANP*	↑	↑	↑	↑
*NPDC1*	↓	↓	↓	↓
*SSH1*	↓	↓	↓	↓
*VGLL4*	↑	↑	↑	↑
*OSM*	↓	↓	↓	↓
*GSDMC*	↓	↓	↓	↓
*MIR21*	↓	↓	↓	↓
*FRMD4A*	↑	↑	↑	↓

Legend: ↑ (red arrow) Hypermethylated; ↓ (blue arrow) Hypomethylated.

**Table 2:** Some examples of DNA methylation changes induced by environmental stressors.

Stressor	Gene/Pathway	Epigenetic Effect (DNA Methylation)	Reference
PM10 (air pollution)	*CD14*	↓ Promoter hypomethylation	Cantone et.al. [92]
PM10 (air pollution)	*TLR4*	↓ Promoter hypomethylation	Cantone et al. [92]
PM10 (metal-rich)	*NOS3*	↓ Hypomethylation correlated with PM and Zn/Fe	Tarantini et al. [93]
PM10 (metal-rich)	*EDN1*	↓ Hypomethylation correlated with Zn exposure	Tarantini et al. [93]
Ozone (O_3_)	*ACE*	↓ Hypomethylation with ozone exposure	Xia et al. [94]
Ozone (O_3_)	*EDN1*	↓ Hypomethylation with ozone exposure	Xia et al. [94]
Cigarette smoke	*AHRR* (cg05575921)	↓ Hypomethylation in smokers	Gutiérrez et al. [95]
Cigarette smoke	*F2RL3* (cg03636183)	↓ Dose-dependent hypomethylation	Zhang et al. [96]
Maternal high-fat diet	*Map2k4*	↓ Hypomethylation in offspring liver	Zhang et al. [97]
Maternal high-fat diet	*Irs2*	↑ Hypermethylation in offspring liver	Zhang et al. [97]
Childhood trauma	*NR3C1*	↑ Promoter hypermethylation	Shields et al. [98]

Legend: ↑ Hypermethylated gene; ↓ Hypomethylated gene.

**Table 3:** Current and platforms and techniques for DNA methylation analysis.

Technique	Conversion Type	Output	Advantages	Limitations
MSP (Methylation-Specific PCR)	Bisulfite	Single gene	Low cost; fast; easy to perform	Harsh conversion; DNA degradation; locus-specific
MS-HRM	Bisulfite	Single gene/small panels	Sensitive; semi-quantitative	Dependent on DNA quality; limited resolution
ddPCR (methylation-based)	Bisulfite	Single gene	High sensitivity and precision	Low multiplexing; DNA degradation
EM-seq	Enzymatic (no bisulfite)	Targeted or genome-wide	Preserves DNA integrity; high-quality data	Higher cost; not standardized
WGBS	Bisulfite	Whole genome	Single-CpG resolution; comprehensive	Very costly; complex analysis
RRBS	Bisulfite	CpG-enriched genome fraction	Lower cost than WGBS; high resolution	Limited genomic coverage
PacBio methylation sequencing	None	Long-read genome-wide	Long reads; native DNA analysis	High cost; infrastructure required
Nanopore methylation sequencing	None	Long-read genome-wide	Direct methylation detection; real-time	Accuracy still improving
Nanopore and Adaptive Sampling	None	Targeted CpG panels	Cost-effective; flexible; rapid turnaround	Protocol optimization needed

## Data Availability

No new data were created or analyzed in this study.
